# Differential roles of the Wip1–p38–p53 DNA damage response pathway in early/advanced-stage ovarian clear cell carcinomas

**DOI:** 10.1186/s12957-022-02600-7

**Published:** 2022-04-30

**Authors:** Chenyang Xu, Takeo Minaguchi, Nan Qi, Kaoru Fujieda, Asami Suto, Hiroya Itagaki, Ayumi Shikama, Nobutaka Tasaka, Azusa Akiyama, Sari Nakao, Hiroyuki Ochi, Toyomi Satoh

**Affiliations:** 1 Doctoral Program in Obstetrics and Gynecology, Graduate School of Comprehensive Human Sciences, University of Tsukuba, 1-1-1 Tsukuba, Ibaraki, 305-8577 Japan; 2 Department of Obstetrics and Gynecology, Faculty of Medicine, University of Tsukuba, 1-1-1 Tsukuba, Ibaraki, 305-8575 Japan

**Keywords:** Wip1, Survival, Advanced stage, Ovarian clear cell carcinoma

## Abstract

**Background:**

Ovarian clear cell carcinoma (OCCC) is one of the most lethal types of ovarian cancer. Early-stage OCCC can be cured by surgery; however, advanced-stage disease shows poor prognosis due to chemoresistance unlike the more common high-grade serous carcinoma.

**Methods:**

We explored the differential roles of the Wip1–p38–p53 DNA damage response pathway in respective early- or advanced-stage OCCC by immunohistochemistry of Wip1, phospho-p38, p53, and phospho-p53 from consecutive 143 patients.

**Results:**

High Wip1 expression correlated with positive p53 (*p*=0.011), which in turn correlated with low nuclear phospho-p38 expression (*p*=0.0094). In the early stages, positive p53 showed trends toward worse overall survival (OS) (*p*=0.062), whereas in the advanced stages, high Wip1 correlated with worse OS (*p*=0.0012). The univariate and multivariate analyses of prognostic factors indicated that high Wip1 was significant and independent for worse OS (*p*=0.011) in the advanced stages, but not in the early stages. Additionally, high Wip1 showed trends toward shorter treatment-free interval (TFI) in the advanced stages, but not in the early stages (*p*=0.083 vs. 0.93). Furthermore, high Wip1 was significantly associated with positive p53 only in the patients with shorter TFI (<6 months), but not in those with longer TFI (≥6 months) (*p*=0.036 vs. 0.34).

**Conclusions:**

Wip1 appears to play a crucial role for the prognosis of OCCC through chemoresistance specifically in the advanced stages, implicating that Wip1 possibly serves as a reasonable therapeutic target for improving chemoresistance and poor prognosis of advanced-stage OCCC.

## Background

Ovarian clear cell carcinoma (OCCC), one of the most lethal types of ovarian cancer, tends to be diagnosed at an early stage which can be cured by surgery. However, advanced-stage OCCC shows poor prognosis due to resistance to platinum-based chemotherapy unlike the more common high-grade serous carcinoma [1–3]. To date, various molecular markers have been suggested to predict prognosis and to serve as possible therapeutic targets in ovarian cancer [4–8]. However, no consensus has been reached yet, especially on specific markers for predicting refractory biological properties of advanced-stage OCCC. DNA damage response is one of the important pathways to ensure genomic integrity. When chronic DNA damage is not repairable, the cells either undergo apoptosis or extend proliferative block. Cells with dysfunctional cell cycle checkpoints and/or apoptotic responses potentially lead to the immortalization of genomic aberrations and tumorigenesis. Prominent events in the early responses induced by DNA damage include the activation of the stress-responsive p38 cascades [9] and the activation of the tumor suppressor p53 [10]. The main function of p38 was confirmed to induce apoptosis [11], and p53 plays a central role in maintaining genomic integrity by preventing the progression of cell cycle or inducing apoptosis after cellular stresses including DNA damage [12]. Wip1 is an oncogene, which is a negative regulator of the p38–p53 signaling pathway through direct and indirect mechanisms. Wip1 directly dephosphorylates and inactivates p53. Wip1 also inactivates p53 through dephosphorylating and inactivating modulators such as p38, Chk1/2, and ATM [13, 14]. Wip1 amplification has been suggested to be associated with a poor prognosis of OCCC [15, 16]. However, the prognostic significance of the Wip1–p38–p53 DNA damage response pathway in OCCC remains to be elucidated. Thus, the aim of our study was to explore the differential prognostic roles of this pathway in early/advanced-stage OCCC. Our findings provide useful information for formulating novel therapeutic strategies for improving the chemoresistance and poor prognosis in advanced-stage OCCC.

## Methods

### Patients and specimens

All patients diagnosed with OCCC who received primary surgery between 1987 and 2016 at the University of Tsukuba Hospital were identified through our database. A total of 143 patients with tumors of pure clear cell histology or mixed histology with clear cell component >50% were included, and the medical records were reviewed. All samples were obtained by the opt-out approach according to the study protocol approved by the Ethics Committee University of Tsukuba Hospital (H26-118). A median follow-up period excluding patients who died was 103 months (range, 7–250 months). Follow-up data were retrieved until 2020-12-1. Overall survival (OS) was defined as the interval between the primary treatment and the last follow-up. Treatment-free interval (TFI) was defined as the interval between the end of the primary adjuvant chemotherapy and recurrence. Staging was conducted based on the criteria of the International Federation of Gynecology and Obstetrics (FIGO, 2014). Treatment of patients was described previously [17]. Table 1 summarizes the patient characteristics.

**Table 1:** Patient characteristics

Patient characteristics	Number (***n***=143)
Median age (range)	54 (30–81)
FIGO stage	
I	82 (57%)
II	20 (14%)
III	28 (20%)
IV	13 (9%)
Histology	
Pure type	134 (94%)
Mixed type	9 (6%)
Serous	4 (3%)
Mucinous	2 (1%)
Endometrioid	2 (1%)
Cystadenocarcinofibroma	1 (1%)
Positive peritoneal cytology	66 (46%)
Endometriosis	
Present	74 (52%)
Absent	69 (48%)
Treatment	
Surgery	143 (100%)
Lymph node adenectomy	112 (78%)
Lymph node sampling	5 (3%)
Chemotherapy	136 (95%)
Platinum	135 (94%)
Taxane	122 (85%)
CPT-11	19 (13%)
Residual tumor present	20 (14%)

*FIGO* International Federation of Gynecology and Obstetrics, *CPT-11* camptothecin-11

### Immunohistochemistry (IHC)

**Fig. 1:**
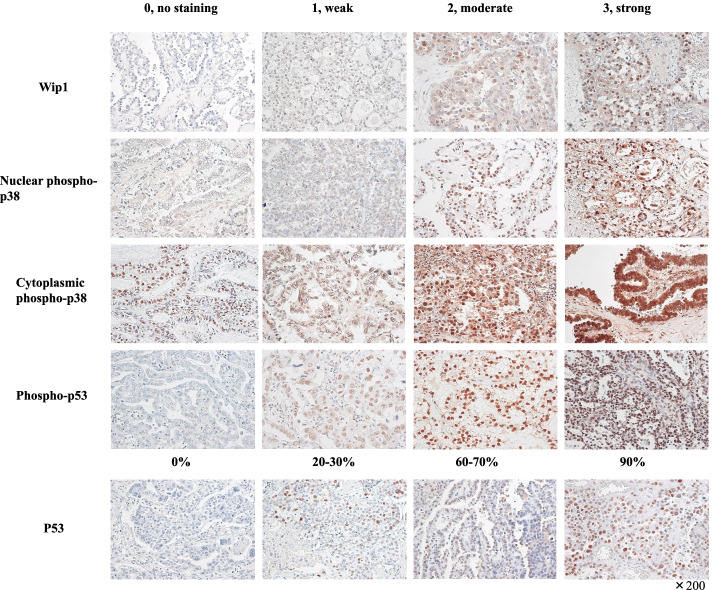
Representative images for immunohistochemical evaluations. The 0 to 3 staining intensity of Wip1, nuclear phospho-p38, cytoplasmic phospho-p38, and phospho-p53 as well as the percentages of nuclear p53 staining. ×200

IHC procedures were described previously [17]. Antibodies used were Wip1 (F-10) (mouse monoclonal, 1:100, Santa Cruz, Dallas, TX, USA), p53 (DO-7) (mouse monoclonal, 1:200, Dako, Tokyo, Japan), phospho-p53 (S15) (rabbit polyclonal, 1:1000, Abcam, Cambridge, UK), and phospho-p38 MAPK (Thr180/Tyr182) (D3F9) (rabbit monoclonal, 1:1000, Cell Signaling, Danvers, MA, USA). The representative images of staining are displayed in Fig. 1 [17].

### IHC scoring

Semiquantitative immunoreactions were assigned by two investigators (CX and TM), and any discrepancies were resolved by conferring over a microscope. For Wip1 and phospho-p53, the nuclear staining was scored by multiplying the percentages of positive tumor cells (0, no positive cell; 1, <10%; 2, 10–50%; and 3, 50%< positive tumor cells) by the most prevalent degree of staining (0, no staining; 1, weak; 2, moderate; and 3, strong). For phosho-p38, nuclear and cytoplasmic staining was separately scored in the same way. P53 staining was evaluated as previously described [17].

### Statistical analyses

Differences in proportions were compared by Fisher’s exact test. Differences in continuous variables were compared by the Mann-Whitney *U* test. The optimal cut-off values of IHC scores for the relationships with OS were determined by the K-Adaptive partitioning method (Table 2) [18]. Survival curves were generated by the Kaplan-Meier method and statistically compared by the log-rank test. The univariate and multivariate analyses were conducted using the Cox proportional hazard model. *P*-values less than 0.05 were considered statistically significant. All statistical analyses were performed using R version 3.5.3.

**Table 2:** The optimal cut-off values of IHC scores for the relationships with OS

	Mean±SD	Stage I/II			Stage III/IV		
		Cut-off	Category	*N* (%)	Cut-off	Category	*N* (%)
Wip1	0.6±1.1	1<	High	18 (18)	1<	High	5 (12)
			Low	84 (82)		Low	36 (88)
P53	8.7±16.8	10%≤	Positive	21 (21)	10%≤	Positive	14 (34)
			Negative	81 (79)		Negative	27 (66)
Phospho-p53	5.3±2.7	2<	High	57 (56)	0<	High	38 (93)
			Low	45 (44)		Low	3 (7)
Nuclear phospho-p38	7.4±1.7	6<	High	50 (49)	5<	High	38 (93)
			Low	52 (51)		Low	3 (7)
Cytoplasmic phospho-p38	3.4±2.8	8<	High	9 (9)	1<	High	30 (73)
			Low	93 (91)		Low	11 (27)

*IHC* immunohistochemical, *OS* overall survival, *SD* standard deviation

## Results

**Fig. 2:**
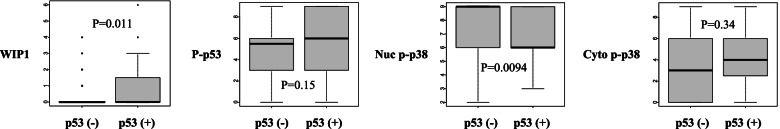
Relationships between the protein expressions and the p53 status in tumors of the whole patients (*n*=143)

**Fig. 3:**
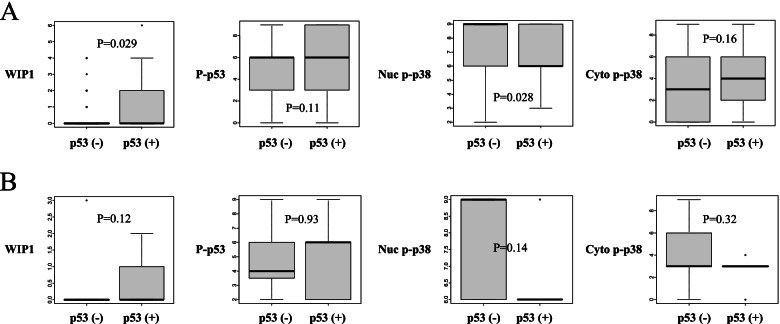
Relationships between the protein expressions and the p53 status in tumors of patients with/without lymph node metastasis. **A** Patients without lymph node metastasis (*n*=123). **B** Patients with lymph node metastasis (*n*=20)

We first examined the relationships among the expressions of Wip1, p53, phospho-p53, and nuclear/cytoplasmic phospho-p38. High Wip1 expression was found to be significantly associated with positive p53 expression, which was significantly associated with low nuclear phospho-p38 expression (*p*=0.011 and 0.0094; Fig. 2). We further examined those relationships separately in the patients with or without lymph node metastasis. High Wip1 was significantly associated with positive p53, which was significantly associated with low nuclear phospho-p38 only in the patients without lymph node metastasis (*p*=0.029 and 0.028; Fig. 3A), but no significant association was found in those with lymph node metastasis (Fig. 3B).

Secondly, we examined the associations between the protein expressions and clinicopathological factors. In the early-stage diseases, positive peritoneal cytology was significantly associated both with low Wip1 expression and with high phospho-p53 expression (*p*=0.011 and 0.017; Table 3). In the advanced-stage diseases, low cytoplasmic phospho-p38 was significantly associated with present residual tumor and showed a trend toward stage IV disease (*p*=0.036 and 0.073, respectively; Table 4). Low phospho-p53 also showed a trend toward present residual tumor (*p*=0.077; Table 4).

**Table 3:** Relationships between the protein expressions and clinicopathological factors in early-stage diseases

Stages I–II (***n***=102)	P53			Phospho-p53			Wip1			Nuclear phospho-p38			Cytoplasmic phospho-p38		
	Positive	Negative		High	Low		High	Low		High	Low		High	Low	
	*n*=21	*n*=81	*p*-value	*n*=57	*n*=45	*p*-value	*n*=18	*n*=84	*p*-value	*n*=50	*n*=52	*p*-value	*n*=9	*n*=93	*p*-value
Age ≥60	4 (19%)	23 (28%)	0.58	13 (23%)	14 (31%)	0.37	7 (39%)	20 (24%)	0.24	13 (26%)	14 (27%)	1	3 (33%)	24 (26%)	0.70
FIGO stage I	17 (81%)	65 (80%)	1	48 (84%)	34 (76%)	0.32	14 (78%)	68 (81%)	0.75	44 (88%)	38 (73%)	0.081	9 (100%)	73 (78%)	0.20
Pure histology	21 (100%)	78 (96%)	1	55 (96%)	44 (98%)	1	17 (94%)	82 (98%)	0.45	49 (98%)	50 (96%)	1	8 (89%)	91 (98%)	0.24
Positive peritoneal cytology	7 (33%)	24 (30%)	0.79	23 (40%)	8 (18%)	0.017	1 (6%)	30 (36%)	0.011	18 (36%)	13 (25%)	0.28	4 (44%)	27 (29%)	0.45
Endometriosis present	13 (62%)	45 (56%)	0.63	37 (65%)	21 (47%)	0.073	13 (72%)	45 (54%)	0.19	29 (58%)	29 (56%)	0.84	5 (56%)	53 (57%)	1
Residual tumor present	0 (0%)	2 (2%)	1	1 (2%)	1 (2%)	1	0 (0%)	2 (2%)	1	1 (2%)	1 (2%)	1	0 (0%)	2 (2%)	1
Lymphadenectmy undone	2 (10%)	13 (16%)	0.73	6 (11%)	9 (20%)	0.26	4 (22%)	11 (13%)	0.30	5 (10%)	10 (19%)	0.26	1 (11%)	14 (15%)	1
Adjuvant chemotherapy performed	19 (90%)	76 (94%)	0.63	53 (93%)	42 (93%)	1	17 (94%)	78 (93%)	1	47 (94%)	48 (92%)	1	8 (89%)	87 (94%)	0.49

*FIGO* International Federation of Gynecology and Obstetrics

**Table 4:** Relationships between the protein expressions and clinicopathological factors in advanced-stage diseases

Stages III–IV (***n***=41)	P53			Phospho-p53			Wip1			Nuclear phospho-p38			Cytoplasmic phospho-p38		
	Positive	Negative		High	Low		High	Low		High	Low		High	Low	
	*n*=14	*n*=27	*p*-value	*n*=38	*n*=3	*p*-value	*n*=5	*n*=36	*p*-value	*n*=38	*n*=3	*p*-value	*n*=30	*n*=11	*p*-value
Age ≥60	6 (43%)	8 (30%)	0.49	13 (34%)	1 (33%)	1	1 (20%)	13 (36%)	0.64	13 (34%)	1 (33%)	1	12 (40%)	2 (18%)	0.28
FIGO stage IV	5 (36%)	8 (30%)	0.73	13 (34%)	0 (0%)	0.54	2 (40%)	11 (31%)	0.64	13 (34%)	0 (0%)	0.54	7 (23%)	6 (55%)	0.073
Pure histology	11 (79%)	24 (89%)	0.39	32 (84%)	3 (100%)	1	5 (100%)	30 (83%)	1	33 (87%)	2 (67%)	0.39	26 (87%)	9 (82%)	0.65
Lymph node metastasis	5 (36%)	15 (56%)	0.33	20 (53%)	0 (0%)	0.23	2 (40%)	18 (50%)	1	20 (53%)	0 (0%)	0.23	16 (53%)	4 (36%)	0.48
Positive peritoneal cytology	12 (86%)	23 (85%)	1	32 (84%)	3 (100%)	1	4 (80%)	31 (86%)	0.57	32 (84%)	3 (100%)	1	26 (87%)	9 (82%)	0.65
Endometriosis present	5 (36%)	11 (41%)	1	15 (39%)	1 (33%)	1	3 (60%)	13 (36%)	0.36	16 (42%)	0 (0%)	0.27	11 (37%)	5 (45%)	0.72
Residual tumor present	5 (36%)	13 (48%)	0.52	15 (39%)	3 (100%)	0.077	2 (40%)	16 (44%)	1	16 (42%)	2 (67%)	0.57	10 (33%)	8 (73%)	0.036
Lymphadenectmy undone	4 (29%)	12 (44%)	0.50	14 (37%)	2 (67%)	0.55	1 (20%)	15 (42%)	0.63	15 (39%)	1 (33%)	1	10 (33%)	6 (55%)	0.29

*FIGO* International Federation of Gynecology and Obstetrics

**Fig. 4:**
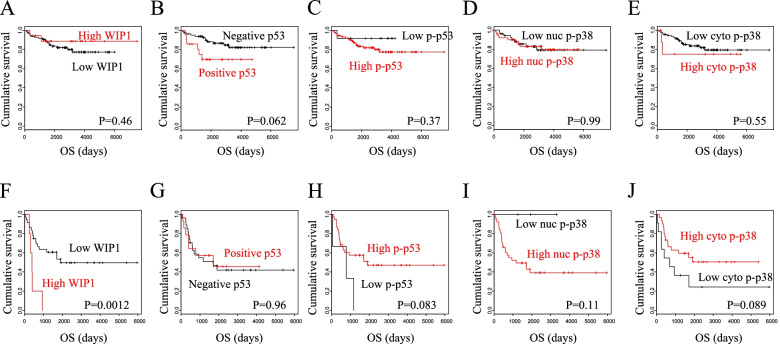
Kaplan-Meier curves for overall survival based on the protein expressions. **A** Patients with early-stage tumors expressing high vs. low Wip1 (*n*=18 vs. 84). **B** Patients with early-stage tumors expressing positive vs*.* negative p53 (*n*=21 vs. 81). **C** Patients with early-stage tumors expressing high vs. low phospho-p53 (*n*=57 vs. 45). **D** Patients with early-stage tumors expressing high vs. low nuclear phospho-p38 (*n*=50 vs. 52). **E** Patients with early-stage tumors expressing high vs. low cytoplasmic phospho-p38 (*n*=9 vs. 93). **F** Patients with advanced-stage tumors expressing high vs. low Wip1 (*n*=5 vs. 36). **G** Patients with advanced-stage tumors expressing positive vs. negative p53 (*n*=14 vs. 27). **H** Patients with advanced-stage tumors expressing high vs. low phospho-p53 (*n*=38 vs. 3). **I** Patients with advanced-stage tumors expressing high vs. low nuclear phospho-p38 (*n*=38 *vs.* 3). **J** Patients with advanced-stage tumors expressing high vs. low cytoplasmic phospho-p38 (*n*=30 vs. 11). OS, overall survival

Next, we compared the patient OS according to the protein expressions. High Wip1 expression showed a significant association with worse OS in the advanced stages, but no significant difference in the early stages (*p*=0.0012 and 0.46, respectively; Fig. 4F, A). Positive p53 showed a trend toward worse OS in the early stages, but no difference in the advanced stages (*p*=0.062 and 0.96, respectively; Fig. 4B, G). As regards phospho-p53, high expression showed a trend toward better OS in the advanced stages (*p*=0.083; Fig. 4H). As for cytoplasmic phospho-p38, high cytoplasmic phospho-p38 showed a trend toward better OS, but no difference in the early stages (*p*=0.089 and 0.55, respectively; Fig. 4J, E).

Subsequently, we performed the univariate and multivariate analyses of various prognostic factors for OS. In the early-stage diseases, positive peritoneal cytology and present residual tumor were found to be significant and independent for poor OS (*p*=0.00043 and 0.0074, respectively; Table 5), whereas in the advanced stages, high Wip1 expression was found to be significant and independent for poor OS (*p*=0.011; Table 6).

**Table 5:** Univariate and multivariate analyses of prognostic factors for OS in early-stage diseases

Stages I–II (***n***=102)	Univariate			Multivariate		
	HR	95% CI	*p*-value	HR	95% CI	*p*-value
High Wip1	0.58	0.13–2.51	0.46	-	-	-
Positive p53	2.48	0.92–6.65	0.071	-	-	-
High phospho-p53	2.43	0.32–18.3	0.39	-	-	-
High nuclear phospho-p38	1.00	0.39–2.51	0.99	-	-	-
High cytoplasmic phospho-p38	1.56	0.36–6.81	0.56	-	-	-
Age ≥ 60	1.70	0.46–3.60	0.64	-	-	-
Positive peritoneal cytology	8.23	2.93–23.2	0.00065	9.25	3.19–26.9	0.00043
Residual tumor present	9.62	1.21–76.3	0.032	21.7	2.28–205.4	0.0074
Endometriosis present	1.07	0.41–2.76	0.89	-	-	-
Lymphadenectmy undone	2.69	0.96–7.56	0.060	-	-	-
Adjuvant chemotherapy performed	1.07	0.14–8.10	0.94	-	-	-
Pure histology	0.29	0.038–2.18	0.23	-	-	-

*OS* overall survival, *HR* hazard ratio, *CI* confidence interval

**Table 6:** Univariate and multivariate analyses of prognostic factors for OS in advanced-stage diseases

Stages III–IV (***n***=41)	Univariate			Multivariate		
	HR	95% CI	*p*-value	HR	95% CI	*p*-value
High Wip1	4.93	1.70–14.3	0.0033	3.98	1.38–11.5	0.011
Positive p53	0.98	0.40–2.40	0.96	-	-	-
High phospho-p53	0.35	0.10–1.21	0.097	-	-	-
High nuclear phospho-p38	7.86E+07	0–Inf	1.00	-	-	-
High cytoplasmic phospho-p38	0.48	0.20–1.14	0.096	-	-	-
Age ≥60	0.46	0.17–1.25	0.13	-	-	-
Positive peritoneal cytology	1.03	0.30–3.48	0.96	-	-	-
Residual tumor present	2.72	1.45–6.48	0.024	2.39	0.98–5.79	0.055
Endometriosis present	0.82	0.34–1.96	0.66	-	-	-
Lymphadenectmy undone	1.62	0.70–3.75	0.26	-	-	-
Adjuvant chemotherapy performed	NA	NA	NA	-	-	-
Pure histology	1.79	0.42–7.66	0.43	-	-	-

*OS* overall survival, *HR* hazard ratio, *CI* confidence interval, *Inf* infinity, *NA* not applicable

**Fig. 5:**
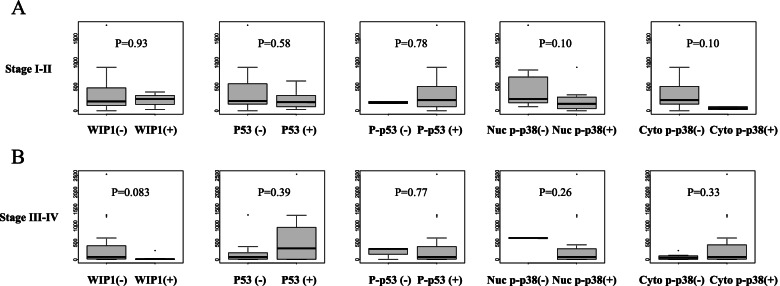
Comparison of treatment-free interval (days) between patients. **A** Early-stage tumors expressing low vs. high Wip1 (*n*=19 vs. 3), negative vs. high p53 (*n*=15 vs. 7), low vs. high phospho-p53 (*n*=2 vs. 20), low vs. high nuclear p38 (*n*=11 vs. 11), and low vs. high cytoplasmic p38 (*n*=20 vs. 2). **B** Advanced-stage tumors expressing low vs. high Wip1 (*n*=24 vs. 5), negative vs. high p53 (*n*=18 vs. 11), low vs. high phospho-p53 (*n*=3 vs. 26), low vs. high nuclear p38 (*n*=1 vs. 28), and low vs. high cytoplasmic p38 (*n*=8 vs. 21)

**Fig. 6:**
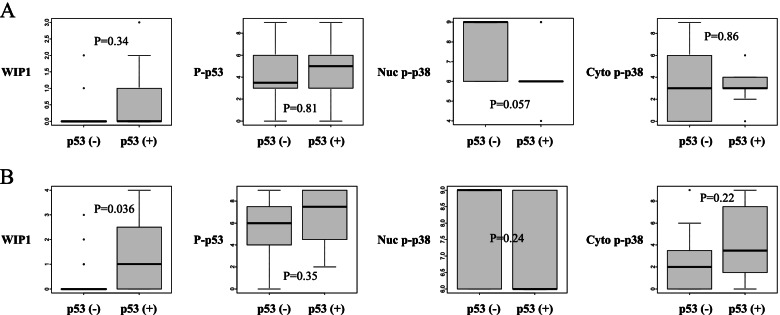
Relationships between the protein expressions and the p53 status in tumors. **A** Patients with treatment-free interval ≥ 180 days (*n*=24). **B** Patients with treatment-free interval < 180 days (*n*=27)

Lastly, we compared TFI according to the protein expressions. Interestingly, positive Wip1 showed a trend toward shorter TFI in advanced stages, but no difference in early stages (*p*=0.083 and 0.93, respectively; Fig. 5). The other proteins showed no association with TFI in either the early or advanced stages (Fig. 5). Furthermore, we examined the relationships between the protein expressions and the p53 status, separately in the patients with longer TFI (≥ 6 months: chemosensitive) and those with shorter TFI (< 6 months: chemoresistant). High Wip1 was significantly associated with positive p53 only in the chemoresistant group, but not in the chemosensitive group (*p*=0.036 and 0.34, respectively; Fig. 6).

## Discussion

Previously, high mRNA expression of *PPM1D*, which encodes Wip1, was reported to significantly correlate with poor survival in OCCC [15, 16]. Recently, abnormal p53 status has been reported to be significantly associated with poor survival in OCCC [19, 20]. Accordingly, the DNA damage response pathway seems to be playing critical roles for the survival of OCCC. Thus, we explored the prognostic significance of the Wip1–p38–p53 axis in the disease. Our IHC analyses indicated that p53 positivity was significantly associated both with high Wip1 expression and with low nuclear phospho-p38 expression (Fig. 2). Wip1 downregulates p53 and nuclear p38 upregulates p53 in the DNA damage response pathway, and positive p53 should correspond to the aberrant inactive protein. Although the detailed mechanism underlying the protein expression profile requires further elucidation, this pathway is suggested to function significantly in the pathogenesis of OCCC.

In our analyses of the associations between the protein expressions and the clinicopathological factors, low cytoplasmic phospho-p38 was found to be significantly associated with present residual tumor and showed a trend toward stage IV disease in the advanced-stage diseases (Table 4). Moreover, patients with low cytoplasmic phospho-p38 showed a trend toward worse OS compared with those with high cytoplasmic phospho-p38 (Fig. 4J). P38 has been reported to activate MMP-2/9 and increase invasive capacity in various types of tumor cells [11, 21]. Therefore, our result may keep in line with this published finding, as upregulation of nuclear p38 may exert tumor progression through transcription, and low cytoplasmic phospho-p38 may correspond to the activated nuclear p38 through nucleocytoplasmic shuttling.

Our survival analyses indicated that patients with positive p53 showed a trend toward worse OS compared with those with negative p53 in the early-stage diseases, while no difference in OS was observed according to p53 status in the advanced-stage diseases (Fig. 4B, G). In our univariate analysis for prognostic factors as well, positive p53 showed a trend toward worse OS in the early-stage diseases in addition to the well-known significant prognostic factors, peritoneal cytology, and residual tumor (Table 5) [22, 23], but not in the advanced-stage diseases (Table 6). Although abnormal p53 status is reported to be associated with poor survival [19, 20], our findings suggest that the prognostic role of p53 in OCCC may be confined to the early-stage diseases.

Patients with high Wip1 showed significantly worse OS compared with those with low Wip1 in the advanced-stage diseases, but not in the early-stage diseases (Fig. 4F, A). Furthermore, the univariate analysis indicated that high Wip1 was a significant factor for poor OS in addition to the well-known prognostic factor, residual tumor in the advanced-stage diseases (Table 6). The subsequent multivariate analysis revealed that high Wip1 was significant and independent for poor OS in the advanced-stage diseases (Table 6). However, no clinicopathologic factor was found to be associated with Wip1 expression in the advanced stages (Table 4). We further compared TFI based on the protein expressions, attempting to identify the underlying mechanism of the prognostic role of Wip1. High Wip1 showed a trend toward shorter TFI in the advanced-stage diseases but not in the early-stage diseases (Fig. 5). Moreover, high Wip1 was significantly associated with positive p53 only in the patients with shorter TFI, but not in those with longer TFI (Fig. 6). These findings suggest that Wip1 may be involved in chemoresistance in the advanced disease of OCCC, as TFI is well known to be an important surrogate marker for the chemosensitivity in ovarian cancer [24–26]. This hypothesis may be supported by the published findings that Wip1 is involved in chemoresistance in other types of cancer cells [27, 28]. Chemoresistance is known to be related with low activity of cellular proliferation. Therefore, our finding that both low Wip1 and high phospho-p53 were significantly associated with positive peritoneal cytology (Table 3) seems consistent with the hypothesis, as Wip1 and its downstream target p53 may be related also with indolent tumor progression. Additionally, our finding that positive p53 was found to be significantly associated both with high Wip1 and with low nuclear phospho-p38 only in the patients without lymph node metastasis, but not in those with lymph node metastasis, also suggests the possible involvement of the Wip1–p38–p53 pathway in indolent tumor progression (Fig. 3).

The present study contains a couple of limitations. First, the retrospective design causes possible selection biases. Second, the evaluation for the protein expressions is based only on the semiquantitative immunohistochemical analysis, and bioinformatics analysis is also lacking. Third, the sample number is relatively small. Nevertheless, our above findings keeping in line with multiple publications may support the validity of our study.

## Conclusions

Early-stage OCCC can be cured by complete surgical resection, while advanced-stage OCCC shows poor prognosis due to the chemoresistance of residual tumor [1–3]. Therefore, our above findings suggest that Wip1 may be a reasonable therapeutic target for improving the poor prognosis of advanced-stage OCCC through enhancing chemosensitivity. A combination of Wip1 inhibitor with chemotherapeutic agents may be useful for advanced-stage OCCC tumors expressing high Wip1. As regards p53-mutated high-Wip1 OCCC, a combination of Wip1 inhibitor with APR-246 [29–31] may be useful by both reactivating mutant p53 and inhibiting the Wip1-mediated downregulation of p53. Further basic and clinical studies are warranted to verify our proposal in order to develop novel strategies for overcoming chemoresistance of OCCC.

## Data Availability

The datasets used and/or analyzed during the current study are available from the corresponding author on reasonable request.

## Abbreviations

FIGO: International Federation of Gynecology and Obstetrics
IHC: Immunohistochemistry
OCCC: Ovarian clear cell carcinoma
OS: Overall survival
TFI: Treatment-free interval
